# Merkel Cell Carcinoma With Divergent Differentiation—Or Something Else? A Triphasic Cutaneous Carcinoma With Lineage Plasticity

**DOI:** 10.1111/cup.70144

**Published:** 2026-05-19

**Authors:** Zeinab Khalil, Hozaifa Elameen, Ahmed K. Alomari

**Affiliations:** ^1^ Department of Pathology and Laboratory Medicine East Carolina University Greenville North Carolina USA; ^2^ Department of Pathology and Laboratory Medicine Indiana University School of Medicine Indianapolis Indiana USA

**Keywords:** Merkel cell carcinoma, sarcomatoid, squamous

## Abstract

Merkel cell carcinoma (MCC) is a rare, aggressive cutaneous neuroendocrine carcinoma that typically arises in sun‐exposed skin of older or immunosuppressed patients. Combined or collision tumors involving MCC and other cutaneous malignancies are uncommon, and tumors exhibiting more than two distinct malignant phenotypes are exceptionally rare. We report a case of a triphenotypic carcinoma in the left upper arm composed of a neuroendocrine carcinoma with Merkel cell features, a poorly differentiated carcinoma with squamous/ductal differentiation, and a sarcomatoid component, each demonstrating distinct morphologic and immunophenotypic profiles. While the findings favor classification as MCC with divergent epithelial and sarcomatoid differentiation, the precise lineage and cell of origin cannot be definitively established, and alternative interpretations are considered and discussed. This unusual case highlights the diagnostic challenge posed by plastic cutaneous carcinomas and underscores the importance of comprehensive histologic evaluation, broad immunohistochemical workup, and multidisciplinary management. We review the relevant literature of cases with comparable histomorphology and discuss the implications for diagnosis, staging, and treatment.

## 1 Introduction

Merkel cell carcinoma (MCC) is an uncommon but highly aggressive primary cutaneous neuroendocrine malignancy. It has an incidence of roughly 0.5–0.7 per 100 000, with a disease‐specific mortality of 33%–46%, exceeding that of melanoma [1, 2]. MCC typically presents as a rapidly growing red or violaceous nodule on sun‐exposed skin. Approximately 80% of MCCs are associated with Merkel cell polyomavirus (MCPyV), which drives oncogenesis and is linked to fewer UV‐signature mutations [3]. Conversely, MCPyV‐negative tumors arise in sun‐damaged skin of older adults, particularly those with chronic ultraviolet (UV) exposure and immunosuppression, and exhibit a high mutational burden, frequently involving the *TP53* and *RB1* genes [3, 4]. Virus‐negative MCCs are also the subset most commonly associated with combined or composite tumors [3].

Combined or collision tumors involving MCC and another cutaneous carcinoma, most often squamous cell carcinoma (SCC), are rare but increasingly recognized [4, 5]. Molecular studies of MCC–SCC tumors have demonstrated shared genetic alterations between components, supporting a common clonal origin and suggesting trans‐differentiation from a conventional epithelial carcinoma [3, 6, 7, 8, 9, 10]. Overlying actinic keratosis or carcinoma in situ is frequently present, further supporting this model [6]. Tumors exhibiting three distinct malignant phenotypes, however, are exceedingly uncommon. Similarly, MCC with sarcomatoid differentiation, essentially a cutaneous carcinosarcoma, is also rare with occasional reported cases [11]. MCC has been reported in collision with basal cell carcinoma [5], adnexal carcinomas [12], and, rarely, melanoma among other neoplasms [13].

Here, we report a highly unusual triphenotypic cutaneous carcinoma composed of a neuroendocrine carcinoma with Merkel cell features, a squamous/ductal carcinoma, and a sarcomatoid carcinoma. While the findings and conventional classification favor a diagnosis of MCC with divergent differentiation, recent studies suggest that, in some combined MCC–SCC tumors, the MCC‐like component may be derived from a squamous/keratinocytic precursor [8, 9, 10]. Therefore this case raises broader questions regarding lineage plasticity in cutaneous carcinoma and the limitations of rigid diagnostic categorization.

## 2 Case

A 79‐year‐old Caucasian woman with a complex oncologic history presented with a rapidly enlarging lesion on the left upper arm. Her medical history was notable for non‐Hodgkin lymphoma in remission following R‐CHOP therapy 12 years earlier. She also had a cutaneous squamous cell carcinoma of the right temple/auricular region diagnosed one year prior, treated with Mohs micrographic surgery followed by adjuvant cemiplimab and palliative radiotherapy for metastatic nodal recurrence. She had received multiple additional courses of radiotherapy to the head and neck for recurrent SCC.

Approximately four months prior to diagnosis, the patient noticed a firm, blue‐black nodule on the left upper arm (Figure 1). On clinical examination two months later, the lesion measured approximately 1.6–1.7 cm, was firm, and appeared violaceous. Given her clinical history and the lesion morphology, the differential diagnosis included nonmelanoma skin cancer, Merkel cell carcinoma, and melanoma. The lesion was subsequently excised with 1‐cm margins.

**FIGURE 1:**
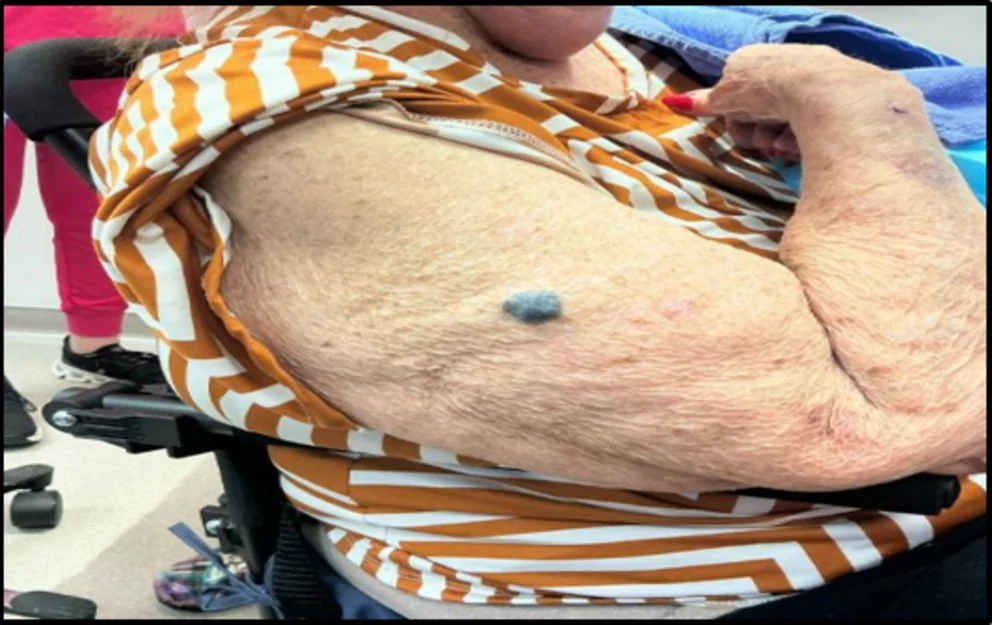
Triphenotypic cutaneous carcinoma. Clinical photograph demonstrating a firm, pigmented, blue‐black nodule on the left upper arm.

Gross examination of the excision specimen revealed a well‐defined, firm mass measuring 2.7 × 2.3 × 0.8 cm. Histopathologic evaluation demonstrated a complex malignant neoplasm composed of three distinct but intimately admixed components (Figure 2). The first component consisted of nests and sheets of small basophilic cells with scant cytoplasm, finely granular (“salt‐and‐pepper”) chromatin, nuclear molding, frequent mitotic figures, and brisk apoptotic activity. This component showed dot‐like CK20 staining and strong expression of neuroendocrine markers, supporting a diagnosis of neuroendocrine carcinoma with Merkel cell features (Figure 3).

**FIGURE 2:**
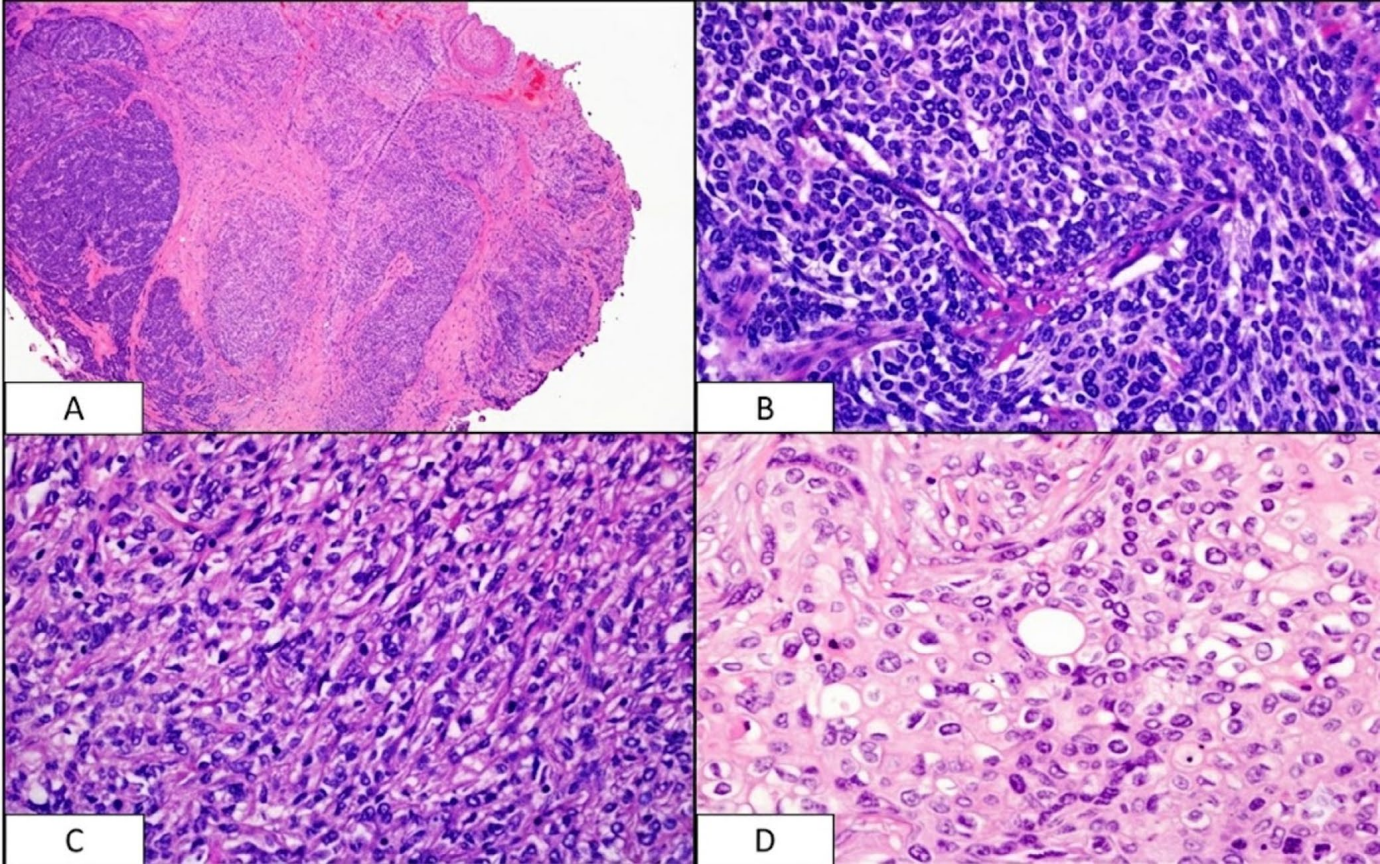
Triphenotypic cutaneous carcinoma. Histopathologic findings showing neuroendocrine, sarcomatoid, and squamous/ductal differentiation on hematoxylin and eosin stain (H&E). (A) Low‐power view showing intimate admixture of the neuroendocrine, sarcomatoid, and squamous/ductal components within the same neoplasm (H&E, 40×). (B) High‐power view of the neuroendocrine component, composed of small blue cells with scant cytoplasm, finely granular chromatin, and brisk mitotic activity (H&E, 400×). (C) High‐power view of the sarcomatoid component, showing spindle‐cell morphology with marked pleomorphism and mitotic activity (H&E, 400×). (D) High‐power view of the squamous/ductal component, showing squamoid nests and duct‐like structures consistent with ductal differentiation (H&E, 400×).

**FIGURE 3:**
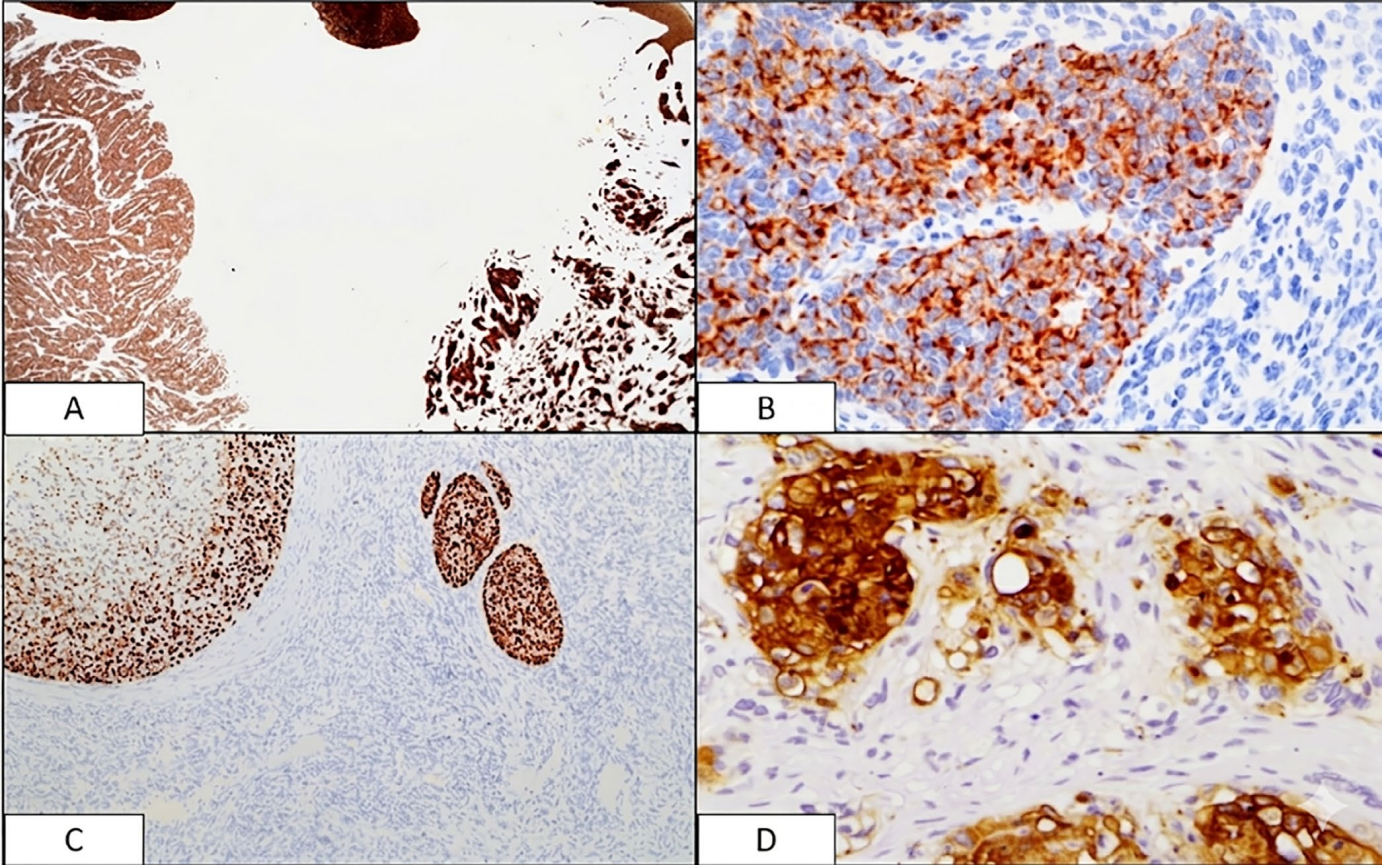
Triphenotypic cutaneous carcinoma. Immunohistochemical findings supporting epithelial, neuroendocrine, and ductal differentiation. (A) Pan‐cytokeratin (AE1/AE3) immunohistochemical stain highlights epithelial differentiation and the distribution of the tumor components (AE1/AE3, 40×). (B) Cytokeratin 20 (CK20) immunohistochemical stain shows dot‐like paranuclear positivity in the neuroendocrine component, with absent staining in the sarcomatoid component (CK20, 400×). (C) p40 immunohistochemical stain highlights squamous differentiation, with negative staining in the sarcomatoid component (p40, 100×). (D) Polyclonal carcinoembryonic antigen (CEA‐poly) immunohistochemical stain highlights ductal differentiation and accentuates duct‐like structures (CEA‐poly, 400×).

A second component demonstrated cohesive nests of polygonal epithelial cells with intercellular bridges, focal ductal lumina, and multifocal connection with an overlying carcinoma in situ. This component expressed high–molecular‐weight cytokeratins and p40, supporting squamous differentiation (Figure 3). Rare duct‐like structures were highlighted by EMA and polyclonal CEA, supporting focal ductal differentiation (Figure 3). Additional CK7 immunohistochemistry demonstrated staining in the squamoid/ductal epithelial component, further supporting adnexal ductal differentiation in this area (Figure 4). Although the morphology was not entirely classic for squamoid eccrine ductal carcinoma, the combined histologic and immunophenotypic findings support a component with squamous and focal ductal/adnexal differentiation. In isolation, diagnostic considerations for this component would include squamoid eccrine ductal carcinoma and eccrine porocarcinoma.

**FIGURE 4:**
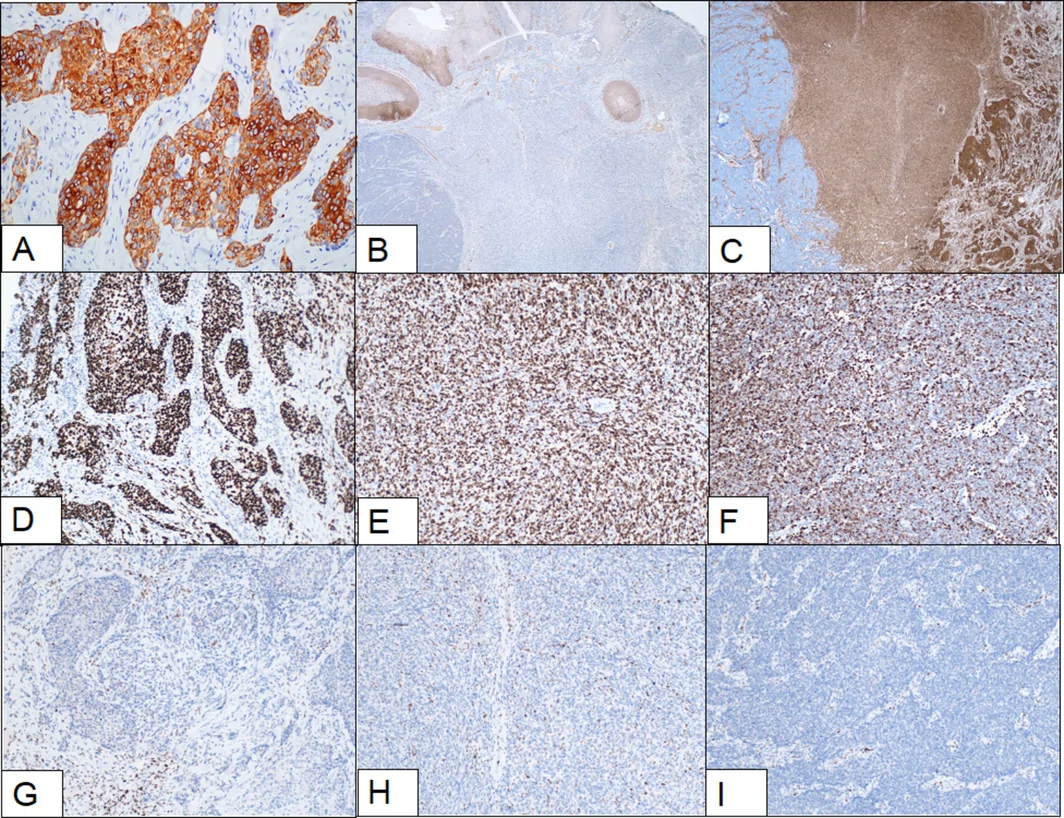
Triphenotypic cutaneous carcinoma. Additional immunohistochemical findings supporting squamous/ductal differentiation and shared aberrant tumor suppressor expression. (A) Cytokeratin 7 (CK7) highlights the squamous/ductal epithelial component, supporting focal adnexal ductal differentiation (CK7, 200×). (B) Nuclear protein in testis (NUT) is largely negative in the tumor, arguing against NUT carcinoma (NUT, 40×). (C) Yes‐associated protein 1 (YAP1) N‐terminus expression is retained in the squamous/ductal and sarcomatoid components but is lost in the neuroendocrine component (YAP1 N‐terminus, 40×). (D–F) p53 shows aberrant overexpression in the squamous/ductal, sarcomatoid, and neuroendocrine components, respectively (p53, 100×, 200×, and 200×, respectively). (G–I) Retinoblastoma protein 1 (RB1) expression is markedly lost in the squamous/ductal, sarcomatoid, and neuroendocrine components, respectively (RB1, 100×, 200×, and 200×, respectively).

The third component was composed of pleomorphic spindle and ovoid cells with marked cytologic atypia and abundant mitotic activity. This component lacked expression of epithelial and neuroendocrine markers, consistent with sarcomatoid dedifferentiation. Lymphovascular invasion was identified, and the microscopic margins were close. Immunohistochemical findings supporting the triphasic differentiation are summarized in Figure 3. The final integrated diagnosis was rendered as a carcinoma with Merkel cell features associated with squamous/ductal and sarcomatoid components.

Following multidisciplinary tumor board discussion, adjuvant radiotherapy and systemic therapy were recommended given the high‐risk features and close margins. However, the patient's course was complicated by unrelated infectious complications and progression of her head and neck squamous cell carcinoma. Despite resumption of immunotherapy, her condition deteriorated and the family ultimately elected palliative care.

## 3 Discussion

This case represents an exceptionally rare triphenotypic cutaneous carcinoma exhibiting neuroendocrine, squamous/ductal, and sarcomatoid differentiation. To our knowledge, only one prior case with comparable trilineage differentiation of MCC, sweat gland carcinoma, and sarcomatoid elements has been reported in the literature [12]. Another report included a case of MCC with dual squamous and sarcomatoid differentiation [7, 14]. Taken together, these reports provide further support that MCC can occasionally arise in concert with divergent epithelial and mesenchymal components, essentially forming a cutaneous carcinosarcoma with a MCC element. It is possible that in such cases a common progenitor cell (pluripotent stem cell) undergoes divergent malignant transformation, giving rise to multiple phenotypes within one tumor [5, 11, 12, 15].

We favor interpretation of this tumor as a Merkel cell carcinoma with divergent epithelial and sarcomatoid differentiation [8, 9, 10]. This classification is supported by the presence of a classic MCC‐like component with characteristic morphology and neuroendocrine immunophenotype, the known propensity of virus‐negative MCC to arise in association with other epithelial carcinomas, the established literature demonstrating shared clonal origins in combined MCC–SCC tumors and the current convention [3, 7]. However, recent studies of combined Merkel cell carcinoma and squamous cell carcinoma have demonstrated substantial molecular overlap between squamous and neuroendocrine components, supporting clonal relatedness and suggesting that, in at least a subset of cases, the MCC‐like component may arise from a squamous dysplastic or keratinocytic precursor rather than representing a purely independent collision phenomenon. Accordingly, although the presence of a classic MCC‐like component with characteristic morphology and neuroendocrine immunophenotype supports classification within the spectrum of Merkel cell carcinoma, the alternative possibility of a highly plastic carcinoma with secondary neuroendocrine transformation should also be acknowledged. This concept is supported by recent genomic studies of combined tumors and should be considered when interpreting unusual triphasic neoplasms such as the present case [8, 9, 10].

Although neuroendocrine differentiation is well recognized in a variety of high‐grade carcinomas, particularly in the setting of dedifferentiation [16], the development of a fully developed MCC‐like phenotype remains rare. In the present case, the presence of an in situ epithelial component raises the additional possibility that this neoplasm may have originated as a primary squamous/ductal carcinoma that subsequently acquired neuroendocrine and sarcomatoid features during tumor progression. This interpretation is also biologically plausible in light of recent literature suggesting squamous‐to‐neuroendocrine evolution in some combined MCC–SCC tumors. Nevertheless, based on current diagnostic convention and the morphologic and immunophenotypic prominence of the MCC‐like component, we believe the most practical classification remains MCC with divergent epithelial and sarcomatoid differentiation, while recognizing that the precise lineage direction cannot be definitively established in this case [8, 9, 10]. With regard to the squamoid/ductal component, we acknowledge that the morphology is not entirely classic for SEDC or porocarcinoma. However, focal duct‐like structures highlighted by EMA and polyclonal CEA, together with CK7 expression in this component, support at least focal adnexal ductal differentiation (Figure 4). Molecular testing for recurrent porocarcinoma‐associated fusions was not performed; therefore, a porocarcinoma‐like molecular signature cannot be confirmed or excluded. For this reason, we have used the descriptive term squamous/ductal epithelial component rather than assigning a definitive diagnosis of SEDC.

Comparative immunohistochemical assessment across the distinct tumor components demonstrated concordant aberrations in p53 and RB1, supporting a shared clonal origin (Figure 4). p53 showed aberrant overexpression in the epithelial/ductal, sarcomatoid, and neuroendocrine components, with relatively reduced intensity in the neuroendocrine component. RB1 expression was markedly lost in all three components. These findings support a biologically related neoplasm with divergent differentiation and lineage plasticity, rather than a collision tumor composed of unrelated neoplastic processes. NUT immunostaining was largely negative, arguing against NUT carcinoma (Figure 4). YAP1 N‐terminal expression was retained in the epithelial/ductal and sarcomatoid components but absent in the neuroendocrine component, suggesting immunophenotypic divergence within the neuroendocrine component (Figure 4). While the concordant p53 overexpression and RB1 loss provide strong immunohistochemical support for a clonal relationship among the tumor components, definitive determination of the directionality of lineage evolution would require component‐specific molecular analysis.

This case underscores the diagnostic pitfalls posed by highly plastic cutaneous carcinomas. Limited biopsy sampling could easily capture only one component, leading to misdiagnosis as a squamous cell carcinoma, an adnexal carcinoma or a dermal sarcoma [11, 14]. Recognition of the full spectrum of differentiation requires generous sampling and a broad immunohistochemical panel [15].

In regard to management, there are no separate standardized treatment guidelines for synchronous or combined MCC and other carcinoma, due to the rarity of such occurrences [4, 7]. From a clinical standpoint, staging and management is guided by the most aggressive component. Hence, in this particular patient and following multidisciplinary discussion, the plan was to treat as Merkel cell carcinoma. Unfortunately, her deteriorating performance status limited further aggressive therapy. An important consideration when performing sentinel lymph node biopsy in patients with collision tumors is to be aware of the different morphologies and the potential spread of only one of the components to the sampled lymph nodes [7].

Combined tumors reported to date appear to behave similarly to stage‐matched virus‐negative MCC, suggesting that biologic behavior is driven more by virus‐negativity (UV‐induced genomic instability) and aggressive features (large size, nodal involvement) than by the mere presence of multiple histologic components [7]. Nevertheless, sarcomatoid differentiation is generally regarded as an adverse prognostic feature in carcinomas, reflecting advanced dedifferentiation and aggressive behavior [16]. Recent literature has also provided genetic evidence supporting true sarcomatoid transformation in MCC, rather than a purely coincidental collision with a separate spindle cell malignancy [17]. In addition, broader recent immunophenotypic studies have shown that sarcomatoid change and squamous atypia may be associated with distinctive marker profiles in MCC, further supporting the morphologic plasticity of these tumors [18]. In a 2013 review, the authors noted that all reported MCC cases with sarcomatous differentiation up to that point had developed lymph node metastases [16]. Likewise, another report of 12 cases of “Merkel cell carcinosarcoma” emphasized the risk of misinterpreting the bland spindle component as reactive stroma [11].

## 4 Conclusion

We report a rare triphenotypic cutaneous carcinoma composed of a neuroendocrine carcinoma with Merkel cell features, a squamous/ductal carcinoma, and a sarcomatoid carcinoma. While the findings favor classification as Merkel cell carcinoma with divergent differentiation, alternative lineage interpretations are worthy of consideration and further investigations [12, 15]. This case highlights the remarkable plasticity of cutaneous carcinomas, the limitations of rigid lineage assignment in highly dedifferentiated tumors, and the importance of comprehensive histologic and immunophenotypic evaluation. Additional molecular characterization of similar cases will be essential to clarify whether these neoplasms represent MCC with divergent differentiation or keratinocytic tumors with secondary neuroendocrine and sarcomatoid transformation [3, 7, 8, 9, 10, 17].

## Author Contributions

Z.K. contributed to manuscript writing, literature review, and editing of images. H.E. contributed to manuscript writing and editing of images. A.K.A. contributed with manuscript writing, editing of images, literature review, and reviewing the final review of the manuscript. All authors have read and agreed to the published version of the manuscript.

## Funding

The authors have nothing to report.

## Consent

The authors have nothing to report.

## Conflicts of Interest

The authors declare no conflicts of interest.

## Data Availability

The data that support the findings of this study are available from the corresponding author upon reasonable request.
